# Elimination of p19^ARF^‐expressing cells protects against pulmonary emphysema in mice

**DOI:** 10.1111/acel.12827

**Published:** 2018-07-30

**Authors:** Ryuta Mikawa, Yohei Suzuki, Hario Baskoro, Kazuki Kanayama, Kazushi Sugimoto, Tadashi Sato, Masataka Sugimoto

**Affiliations:** ^1^ Research Institute National Center for Geriatrics and Gerontology Obu Japan; ^2^ Department of Respiratory Medicine Juntendo University School of Medicine Tokyo Japan; ^3^ Department of Clinical Nutrition Suzuka University of Medical Science Suzuka Japan; ^4^ Department of Molecular and Laboratory Medicine, Department of Gastroenterology Mie University Graduate School of Medicine Tsu Japan; ^5^ Department of Aging Research Nagoya University Graduate School of Medicine Nagoya Japan

**Keywords:** cell ablation, cellular senescence, p19^ARF^, pulmonary emphysema, SASP, senolytic drug

## Abstract

Senescent cells accumulate in tissues during aging and are considered to underlie several aging‐associated phenotypes and diseases. We recently reported that the elimination of p19^ARF^‐expressing senescent cells from lung tissue restored tissue function and gene expression in middle‐aged (12‐month‐old) mice. The aging of lung tissue increases the risk of pulmonary diseases such as emphysema, and cellular senescence is accelerated in emphysema patients. However, there is currently no direct evidence to show that cellular senescence promotes the pathology of emphysema, and the involvement of senescence in the development of this disease has yet to be clarified. We herein demonstrated that p19^ARF^ facilitated the development of pulmonary emphysema in mice. The elimination of p19^ARF^‐expressing cells prevented lung tissue from elastase‐induced lung dysfunction. These effects appeared to depend on reduced pulmonary inflammation, which is enhanced after elastase stimulation. Furthermore, the administration of a senolytic drug that selectively kills senescent cells attenuated emphysema‐associated pathologies. These results strongly suggest the potential of senescent cells as therapeutic/preventive targets for pulmonary emphysema.

## 1 INTRODUCTION

Cellular senescence plays a central role in tumor suppression by halting the proliferation of cells at risk of malignant transformation (Campisi & d’Adda, 2007; Collado, Blasco, & Serrano, 2007). Senescence is executed by the activation of two major tumor suppressor pathways, the p19^ARF^ (p14^ARF^ in human)‐p53 and p16^INK4a^‐pRB pathways, which play critical roles in the induction and maintenance of cell cycle arrest during senescence (Ben‐Porath & Weinberg, 2005; Sherr, 2001). In addition to cell proliferation arrest, it is now evident that senescent cells secrete many pro‐inflammatory factors, proteinases, and other bioactive substances (Coppé et al., 2008). These substances are collectively called SASPs (senescence‐associated secretory phenotypes), through which senescent cells affect the functions of their surrounding nonsenescent cells.

Senescent cells accumulate in several tissues during aging in humans and mice (Dimri et al., 1995; Krishnamurthy et al., 2004) and are considered to contribute to tissue aging and aging‐associated disorders through their cell nonautonomous functions (Freund, Orjalo, Desprez, & Campisi, 2010; Watanabe, Kawamoto, Ohtani, & Hara, 2017). Recent studies using transgenic mice designed to eliminate senescent cells from tissues by sensitizing them to specific drugs have more clearly elucidated the roles of senescent cells in tissue aging and disease. The targeting of senescent cells through a semi‐genetic approach extends the health span by ameliorating the aging‐associated phenotypes of kidney, eye, heart, bone, and lung tissues in wild‐type or progeria model mice (Baar et al., 2017; Baker et al., 2016; Farr et al., 2017; Hashimoto et al., 2016). In addition, senescent cell elimination alleviates pathologies in disease models, which include those of atherosclerosis, idiopathic pulmonary fibrosis (IPF), hepatic steatosis, and osteoarthritis (Childs et al., 2016; Jeon et al., 2017; Ogrodnik et al., 2017; Schafer et al., 2017). As the removal of senescent cells has beneficial effects on the maintenance of tissue homeostasis and the prevention of diseases in mice, senescent cells are expected to have potential as therapeutic targets for these diseases. One possible approach to apply the findings obtained from mouse studies to humans is the pharmacological targeting of senescent cells. Senescence shows resistance to cytotoxic stress due to an enhanced pro‐survival pathway (Wang, 1995); however, certain drugs have the ability to induce cell death in senescent cells by targeting specific signaling pathways (Zhu et al., 2015). The efficacy of drugs that induce senescent cell death, namely senolytic drugs, has been demonstrated in several mouse disease models. The combination of dasatinib and quercetin, which inhibits pro‐survival kinase pathways, ameliorates cardiovascular and vasomotor functions (Roos et al., 2016; Zhu et al., 2015), hepatic steatosis (Ogrodnik et al., 2017), and IPF (Schafer et al., 2017), similar to the semi‐genetic elimination of senescent cells. The anti‐apoptotic bcl‐2 family protein inhibitors ABT‐263/737 are effective in atherosclerosis and osteoarthritis models (Childs et al., 2016; Jeon et al., 2017) and improve stem cell functions and other aging‐associated phenotypes (Chang et al., 2016; Yosef et al., 2016).

Pulmonary emphysema is characterized by the destruction of alveolar walls, leading to permanent enlargement of the airspace in the lungs, and is a major component of chronic obstructive pulmonary disease (COPD), which is currently one of the leading causes of death worldwide. Pulmonary emphysema is also associated with the infiltration of inflammatory cells, which are believed to cause the accumulation of proteinases and lead to alveolar destruction (Barnes, 2004). Macrophages are predominant inflammatory cells in lung tissues, and their number increases in emphysema (Rodriguez, White, Senior, & Levine, 1977). Matrix metalloproteinase (MMP)‐12, also known as macrophage elastase, is required for the development of cigarette smoke‐induced emphysema in mice (Hautamaki, Kobayashi, Senior, & Shapiro, 1997), and the inhibition of macrophage recruitment or induction of apoptosis in alveolar macrophages was found to suppress alveolar collapse in an elastase‐induced emphysema model (Houghton et al., 2006; Ueno et al., 2015). Neutrophils are also known to contribute to the pathology of emphysema, and the inactivation of neutrophil elastase was shown to confer resistance in a mouse emphysema model (Shapiro et al., 2003). Emphysema is accompanied by an increase in cellular senescence (Tsuji, Aoshiba, & Nagai, 2006). However, it currently remains unclear whether and how senescent cells are involved in the development of pulmonary emphysema or whether they are a consequence of this disease.

ARF‐DTR mice express the diphtheria toxin receptor (DTR) and luciferase genes under the control of the *CDKN2A* promoter/enhancer and have enabled us to eliminate p19^ARF^‐expressing cells from lung tissues (Hashimoto et al., 2016). p19^ARF^ accumulates in mesenchymal cells of the lung parenchyma in adult mice, and the removal of p19^ARF^‐expressing cells was found to restore lung function with concomitant changes in the expression of senescence‐associated genes. Therefore, p19^ARF^‐expressing senescent cells contribute, at least in part, to pulmonary hypofunction, which increases the risk of emphysema. Using ARF‐DTR mice, we herein demonstrate that p19^ARF^‐expressing cells facilitate emphysema‐associated lung dysfunction. The elimination of p19^ARF^‐expressing cells by a toxin‐mediated cell knockout system (Saito et al., 2001) from lung tissue protected against elastase‐induced emphysema. Alveolar wall destruction was reduced, and pulmonary function was maintained, in the absence of p19^ARF^‐expressing cells. The accumulation of inflammatory cells was also suppressed after the elimination of p19^ARF^‐expressing cells, as exemplified by the reduced infiltration of macrophages and other cells after elastase challenge. Moreover, the administration of a senolytic drug conferred resistance to elastase‐induced pulmonary dysfunction with a concomitant reduction in senescent cells in lung tissues. Collectively, the present results imply that p19^ARF^‐expressing cells exacerbate pulmonary emphysema and have potential as a therapeutic/preventive target for emphysema.

## 2 RESULTS

**Figure 1:**
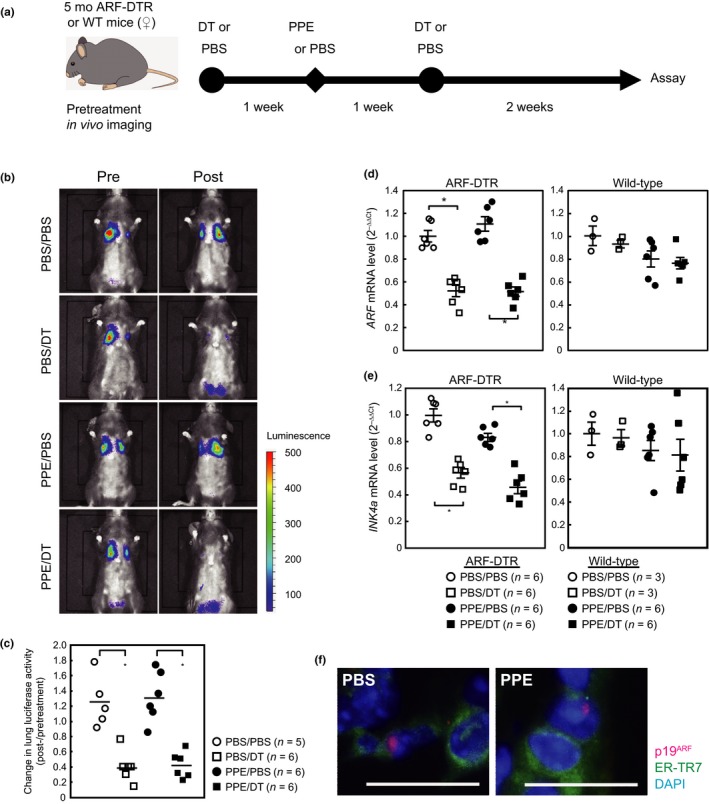
DT eliminated p19^ARF^‐expressing cells from the lung tissues of ARF‐DTR mice. (a) Experimental procedure. Five‐month‐old female ARF‐DTR or wild‐type mice pretreated with PBS or DT were administered porcine pancreatic elastase (100 U/kg). DT and PPE were administered intraperitoneally and intranasally, respectively. (b) Representative images of the in vivo imaging of ARF‐DTR mice. Images were taken before (pre) and after (post) the administration of DT and PPE, as shown in panel a. (c) Luciferase activity was measured using in vivo imaging analysis software. Changes in the luciferase activity before and after drug administration in each mouse were plotted. Bars indicate the average values of each group. (d,e) The total RNA extracted from lung tissue was analyzed by real‐time PCR for the expression of *ARF* (d) or *INK4a* (e). *GAPDH* mRNA was used as an internal standard in each sample, and the ∆∆*C*_t_ method was used to determine the relative expression level. Bars represent the mean ± *SEM*. Data were analyzed by a one‐way ANOVA and Steel–Dwass post hoc analysis. *p < 0.05. (f) Representative images of lung sections of control (PBS) and PPE‐treated mice immunostained using p19^ARF^ (*red*) and fibroblast marker ER‐TR7 (*green*). Sections were counterstained with DAPI (*blue*). Bar; 20 μm

To investigate the roles of p19^ARF^‐expressing cells in the development of pulmonary emphysema, we employed a porcine pancreatic elastase (PPE)‐induced emphysema model that is closely related to human panlobular emphysema. Five‐month‐old female wild‐type or ARF‐DTR mice pretreated with DT or PBS were administered with PPE or PBS for 3 weeks (Figure 1a). ARF‐DTR mice carry extra *CDKN2A* alleles as a transgene, in which the first exon of the *ARF* gene is replaced with DTR (human *HB‐EGF I117V/L148V*) and *firefly* luciferase genes, thereby enabling the elimination and detection of p19^ARF^‐expressing cells by the administration of DT and in vivo imaging, respectively (Hashimoto et al., 2016). An in vivo imaging analysis of lung luciferase activity before and after treatment revealed that the PPE treatment did not affect luciferase activity, whereas it was abolished by DT treatment in the control and PPE‐treated animals (Figure 1b,c). Consistent with these results, *ARF* and *INK4a* mRNA levels in lung tissues were both reduced in DT‐treated ARF‐DTR mice but not in wild‐type mice, although PPE caused no detectable changes in the *ARF* or *INK4a* expression levels by itself (Figure 1d,e) or in the distribution of p19^ARF^, which was observed in cells expressing fibroblast marker (Figure 1f). These results clearly indicate that p19^ARF^‐expressing cells were successfully eliminated from the lung tissues of ARF‐DTR mice, and PPE did not markedly affect the number of these cells observed during the time course examined.

**Figure 2:**
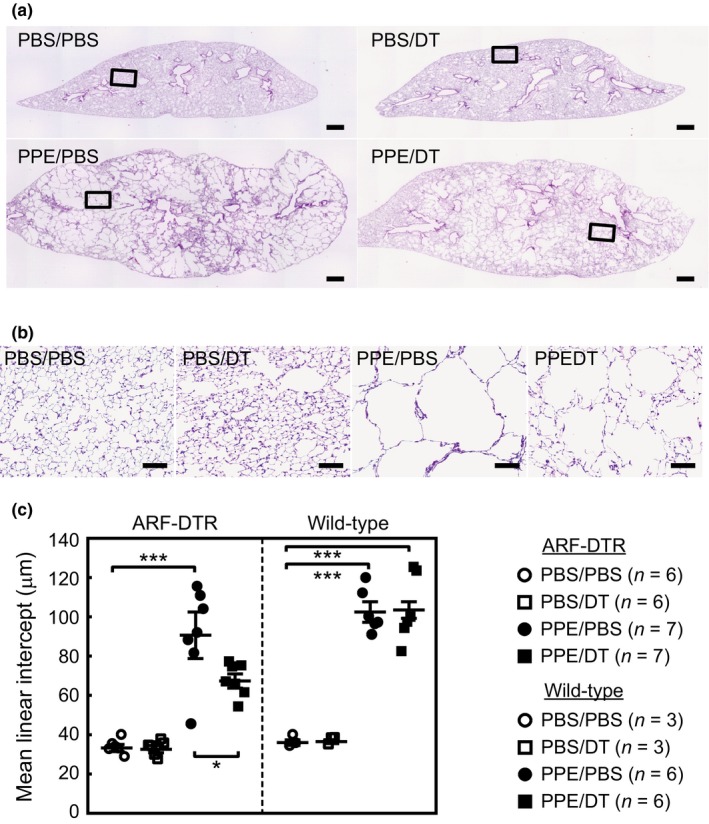
Elimination of p19^ARF^‐expressing cells diminished elastase‐induced alveolar collapse. (a) Lung tissues of mice treated as shown in Figure 1a were fixed at 25 cmH_2_O and subjected to hematoxylin and eosin staining. Representative images are shown. Bar; 500 μm. (b) Representative enlarged images of mouse lung sections. The area indicated in panel A was enlarged. Bar; 100 μm. (c) Alveolar mean linear intercepts were measured. At least 300 alveoli were counted in each mouse. Bars represent the mean ± *SEM*. Data were analyzed by a one‐way ANOVA and Tukey–Kramer post hoc analysis. *p < 0.05 and ***p < 0.001

Pulmonary emphysema is characterized by an enlarged airspace as a result of irreversible alveolar collapse. We examined the impact of p19^ARF^‐expressing cell elimination on emphysema‐associated morphology in these mice. Lung tissues were inflated with fixative solutions under constant pressure before embedding and sectioning. The PPE treatment caused massive alveolar collapse, and the pre‐elimination of p19^ARF^‐expressing cells attenuated this effect (Figure 2a,b). The results of a morphometric analysis indicated that the alveolar mean linear intercept, which reflects the mean alveolar size, increased threefold in PPE‐treated animals due to alveolar collapse, and this result was suppressed by approximately 50% in DT‐treated animals (Figure 2c). The effects of DT are attributed to the elimination of p19^ARF^‐expressing cells because DT had no effect on PPE‐treated wild‐type mice. DT had no significant effects on the PPE‐untreated ARF‐DTR mice at this age (5 months old) but restored alveolar walls and their size in older animals (Hashimoto et al., 2016).

**Figure 3:**
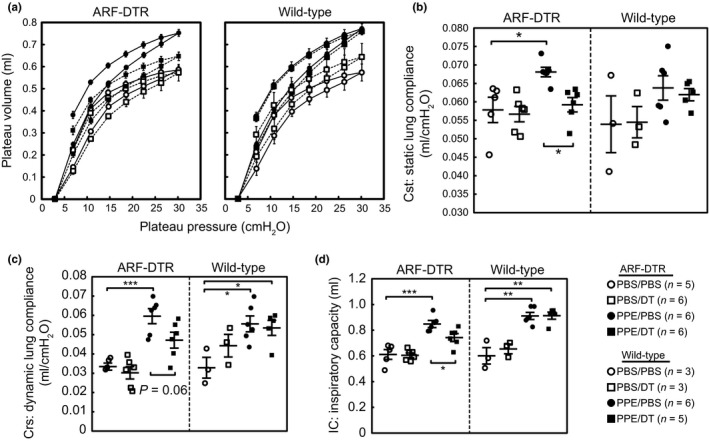
Elimination of p19^ARF^‐expressing cells protected lung tissues from elastase‐induced pulmonary dysfunction. (a) Pressure–volume curves of ARF‐DTR (*left*) and wild‐type (*right*) mouse lungs treated as shown in Figure 1a. Mice in the indicated groups were euthanized and connected to the FlexiVent system through their tracheae. Mice were subjected to laparotomy, and the diaphragm was removed prior to the assay. Data represent the means ± *SEM*. (b–d) Static lung compliance (Cst), dynamic lung compliance (Crs), and inspiratory capacity (IC) are shown. Cst was calculated from the slope of the pressure–volume loop and reflects the static elastic recoil pressure of tissues at a given lung volume. Crs was obtained using the single frequency forced oscillation technique and represents pulmonary compliance during the periods of gas flow. Bars represent the mean ± *SEM*. Data were analyzed by a one‐way ANOVA and Tukey–Kramer post hoc analysis. *p < 0.05, **p < 0.01 and ***p < 0.001

We then attempted to clarify whether the physiological function of lung tissues is retained after PPE treatment by eliminating p19^ARF^‐expressing cells. We performed pulmonary function tests using spirometry. The PPE treatment significantly increased both static and dynamic lung tissue compliance (Cst and Crs, respectively) as well as total lung capacity (inspiratory capacity; IC) due to alveolar collapse (Figure 3). However, the effects of DT on lung function were diminished in mice pretreated with DT, and PPE exerted weaker effects on Cst, Crs, and IC in DT‐treated ARF‐DTR mice than in wild‐type mice. Collectively, these results strongly suggest that the elimination of p19^ARF^‐expressing cells has protective effects against PPE‐induced lung dysfunction. Nevertheless, DT did not affect all of the observed PPE‐induced changes in the parameters examined (Figure S1), suggesting that the effects of p19^ARF^‐expressing cell elimination are limited in the PPE‐induced lung injury model. Similar results were obtained in male ARF‐DTR mice (data not shown).

We also tested the effects of p19^ARF^‐expressing cell elimination in older (13–14 months old) female animals. Lung compliance was decreased by DT treatment in the absence of PPE treatment in those mice (Figure S2). PPE increased the lung compliance (Crs), which was ameliorated by DT treatment in ARF‐DTR but not in wild‐type animals. These results likely reflect our previous observation that lung function can be restored by eliminating the p19^ARF^‐expressing cells in aged (12 months old or older) animals (Hashimoto et al., 2016). To focus on the effects of p19^ARF^‐expressing cells on PPE‐induced lung pathology, we used 5‐month‐old mice for further analysis.

**Figure 4:**
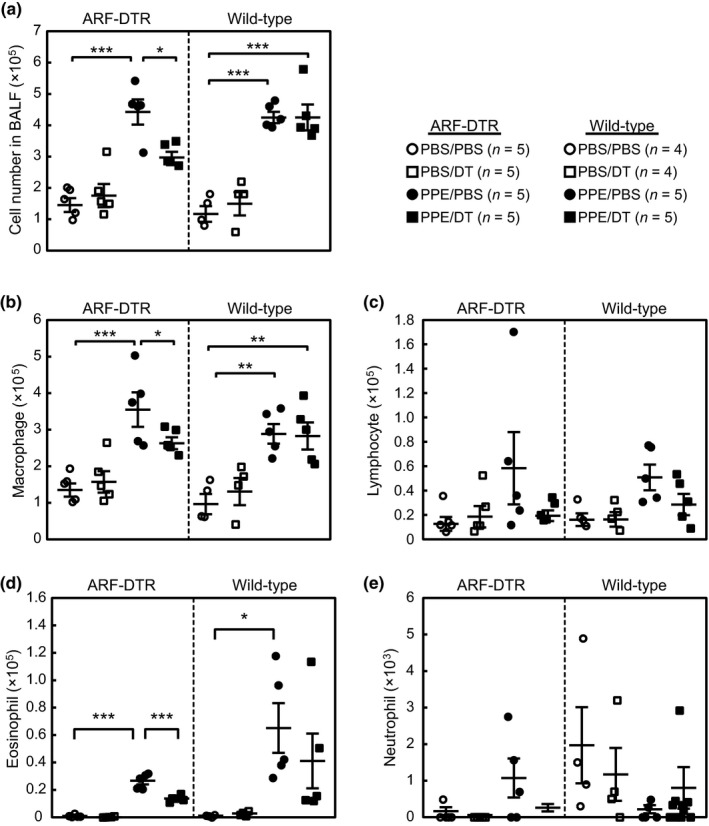
Elimination of p19^ARF^‐expressing cells suppressed the accumulation of inflammatory cells after the elastase treatment. (a) Bronchoalveolar lavage fluid (BALF) was prepared from mice treated with elastase for 1 week, and the total cell number in BALF was counted. (b–e) BALF cells were centrifuged onto glass slides and stained by modified Giemsa staining. The numbers of macrophages (b), lymphocytes (c), eosinophils (d), and neutrophils (e) in each BALF sample were counted. Bars represent the mean ± *SEM*. Data were analyzed by a one‐way ANOVA and Tukey–Kramer post hoc analysis. *p < 0.05 and ***p < 0.001

To gain further insight into the role of p19^ARF^‐expressing cells in PPE‐induced emphysema, we analyzed cells in bronchoalveolar lavage fluid (BALF). Although a significant change was observed in lung morphology and function 3 weeks after the administration of PPE (Figures 2 and 3), no significant difference was found in the number or composition of inflammatory cells in BALF among PPE‐ and/or DT‐treated samples at this time point (Figure S3). This result was expected because a single shot of PPE only has temporal effects on BALF cells in the C57BL/6J strain (Limjunyawong, Craig, Lagassé, Scott, & Mitzner, 2015; Ueno et al., 2015). Therefore, we analyzed BALF cells at an earlier time point after the administration of PPE. Mice were treated with DT and PPE, and BALF cells were collected 1 week after the administration of PPE (Figure 1a). In contrast to the 3‐week treatment, a significant increase was noted in the total cell number in the BALF of PPE‐treated lungs at this time point (Figure 4a). This increase largely accounted for the change in macrophage numbers (Figure 4b), although the number of other inflammatory cells was also slightly increased (Figure 4c–e). The DT treatment diminished these effects, and the increase in inflammatory cells by PPE was significantly suppressed in ARF‐DTR but not in wild‐type mice. Taken together, these results suggest that the presence of p19^ARF^‐expressing cells in the lungs facilitates PPE‐induced inflammation.

Macrophages have been reported to express high levels of p16^INK4a^ and senescent‐associated *β*‐galactosidase activity (Hall et al., 2017). Although p19^ARF^ expression in macrophages has not been documented, and no luciferase activity was detected in the BALF cells of ARF‐DTR mice (Hashimoto et al., 2016), it is still possible that DT acted through the macrophage depletion in PPE‐treated ARF‐DTR mice. We checked the expression of *INK4a*, *ARF,* and *DTR/Luc* in BALF cells prepared from ARF‐DTR mice treated with PPE for 1 week. Although very low levels of *INK4a* were detected, both *ARF* and *DTR/Luc* were barely detectable in these cells (Figure S4). Moreover, the *INK4a* level was unchanged in the BALF cells of DT‐treated mice. Thus, it is unlikely that the effects of DT on PPE‐induced emphysema were attributed to the ablation of nonsenescent macrophages in ARF‐DTR mice.

**Figure 5:**
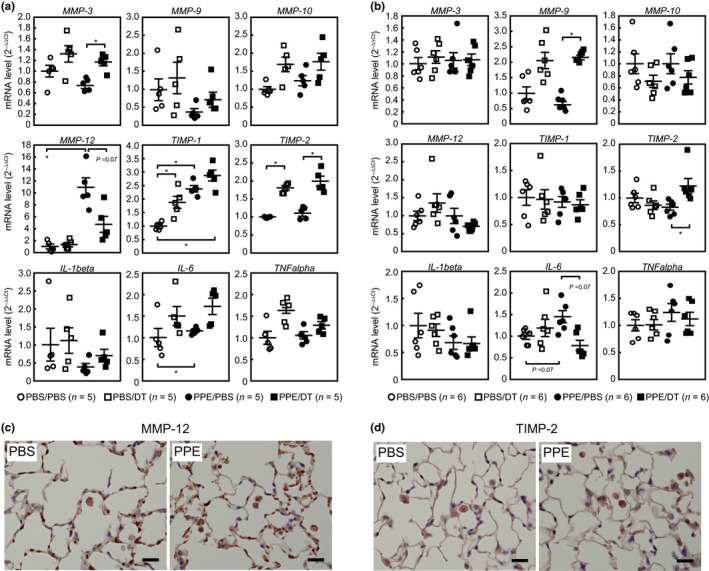
Effects of PPE/DT administration on SASP‐related factors. (a,b) Female ARF‐DTR mice were treated with PPE and/or DT as shown in Figure 1a. The total RNA was isolated from lung tissues 1 (a) or 3 weeks (b) after the administration of PPE. The expression of the indicated genes was analyzed by real‐time PCR. mRNA levels were normalized to *GAPDH* in each sample. Bars represent the mean ± *SEM*. Data were analyzed by a one‐way ANOVA and Steel–Dwass post hoc analysis. *p < 0.05. (c,d) Lung sections of mice treated with PBS/PBS (PBS) or PPE/PBS (PPE) for 1 week were immunostained using MMP‐12 (c) or TIMP‐2 (d) antibody. Sections were counterstained with hematoxylin. Bar; 20 μm

The results described above imply that the elimination of p19^ARF^‐expressing cells suppressed the accumulation of inflammatory cells in lung tissues, thereby protecting these tissues from PPE‐induced emphysema. These inflammatory cells express enzymes that exhibit elastolytic activity. MMP‐12, also known as macrophage elastase, is involved in the development of emphysema, and a targeted deletion of *MMP‐12* confers resistance to cigarette smoke‐induced emphysema (Hautamaki et al., 1997). Furthermore, many pro‐inflammatory cytokines and MMPs are incorporated in SASP in humans and mice (Freund et al., 2010) and are elevated in senescent cells as well as in aged tissue. Therefore, we analyzed the mRNA expression levels of SASP‐related cytokines and MMPs in PPE‐treated lung tissues. Only *MMP‐12* showed a significant increase 1 week after the PPE treatment, and this was inhibited by DT pretreatment (Figure 5a). In addition, we observed an increase in the *tissue inhibitor of metalloproteinase‐2* (*TIMP‐2*) levels in DT‐treated lungs, although PPE by itself had no effect. The effects of DT are attributed to the elimination of p19^ARF^‐expressing cells because DT had no significant effect on PPE‐treated wild‐type animals (Figure S5a). Immunohistochemical analyses revealed that MMP‐12 was expressed in alveolar macrophages and capillary endothelia, although TIMP‐2 was predominantly observed in macrophages (Figure 5c,d). As alveolar fibroblasts are the major p19^ARF^‐expressing cells in the lung (Figure 1f; Hashimoto et al., 2016), these cells likely affected the expression of MMP‐12 and TIMP‐2 cell nonautonomously through SASP. Consistent with previous findings (Limjunyawong et al., 2015; Ueno et al., 2015), the expression of these genes was mostly diminished after 3 weeks, and DT also had a minor effect on these genes at this time point (Figures 5b and S5b). Collectively, these results suggest that the presence of p19^ARF^‐expressing cells in lung tissues facilitates the accumulation of inflammatory cells during the early phase of a PPE challenge, which promotes the development of emphysema‐associated pathologies.

**Figure 6:**
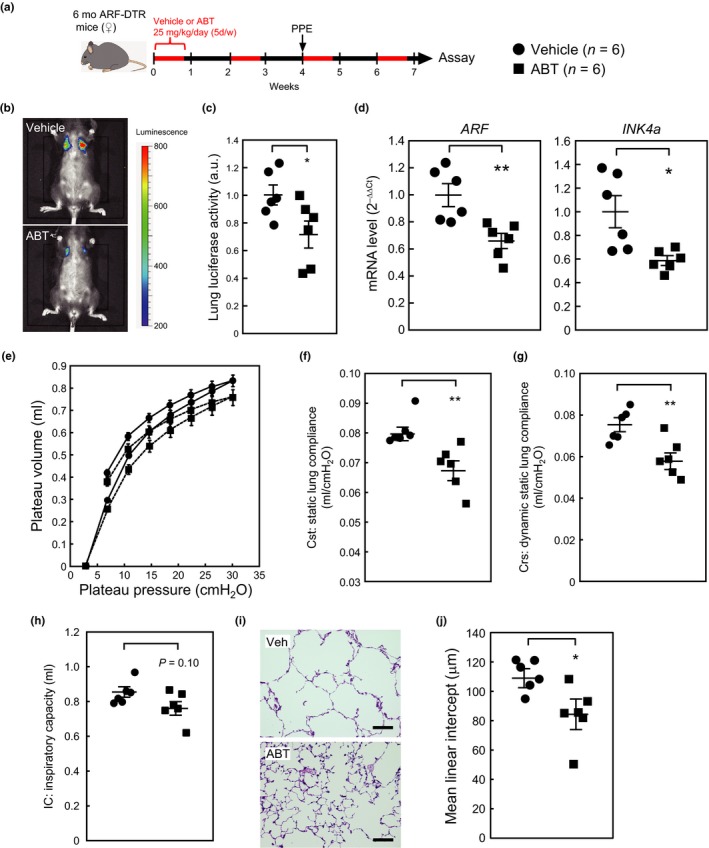
ABT‐263 protected lung tissues from PPE‐induced emphysema. (a) Experimental timeline. Six‐month‐old female ARF‐DTR mice were orally administered ABT‐263 (25 mg kg^−1^ day^−1^) or vehicle alone. PPE was administered intranasally 4 weeks after the first ABT‐263 treatment. (b) Representative images of the in vivo luciferase analysis. (c) Luminescence in the chest region was quantified. Values were normalized to the average value of the vehicle‐treated group. (d) The total RNA extracted from the lung tissues was analyzed by real‐time PCR for the expression of *ARF* (*left*) or *INK4a* (*right*). mRNA levels were normalized to *GAPDH* in each sample. (e–h) Mice were subjected to pulmonary function tests. Pressure–volume loop (e), Cst (f), Crs (g), and IC (h) are shown. (i) Representative images of vehicle‐ or ABT‐263‐treated mouse lung sections. Sections were stained with hematoxylin and eosin. Bar; 100 μm. (j) Alveolar mean linear intercepts were measured. Bars represent the mean ± *SEM*. Data were analyzed by an unpaired Student's *t* test. *p < 0.05, **p < 0.01

These results imply that the pre‐elimination of p19^ARF^‐expressing cells from adult lung tissues has protective effects in a mouse emphysema model. However, the method we utilized to eliminate p19^ARF^‐expressing cells depends entirely on the transgene and is not directly applicable to humans. One alternative approach that may be applicable to humans is “senolytic” agents that selectively kill senescent cells by targeting senescent cell‐specific pro‐survival pathways (Kirkland & Tchkonia, 2015). Several potential senolytic drugs have recently been discovered or developed, and we used ABT‐263 (Navitoclax) in this study. Six‐month‐old female ARF‐DTR mice were orally administered ABT‐263 for 8 weeks, as shown in Figure 6a. An in vivo imaging analysis revealed less luciferase luminescence in the ABT‐263 group than in the control (vehicle) group (Figure 6b,c); however, this effect may be weaker than that of DT. Nevertheless, the *ARF* and *INK4a* levels were both down‐regulated by ABT‐263 (Figure 6d), suggesting that p19^ARF^‐ and p16^INK4a^‐expressing cells were at least partially eliminated from lung tissues.

We then performed pulmonary function tests on these mice. The administration of ABT‐263 restored lung tissue elasticity in PPE‐treated animals, as represented by decreased lung tissue compliance (Figure 6e–g). Other parameters, including IC, Rrs, G, and H, were also ameliorated in the ABT‐263‐treated group (Figures 6h and S6). However, ABT‐263 did not affect Rn, which appears to reflect the fact that the elimination of p19^ARF^‐expressing cells has no influence on central airway resistance in aged animals (Hashimoto et al., 2016) or in a PPE‐induced emphysema model (Figure S1). Consistent with these results, a morphometric analysis of lung sections revealed that ABT‐263 partly restored alveolar size (mean linear intercept) in these mice (Figure 6i,j). Collectively, these results imply that senolysis prevents aspects of chemically induced emphysema.

## 3 DISCUSSION

Pulmonary emphysema is a progressive lung disease characterized by the permanent enlargement of airspaces and is one of the most common conditions of COPD. Emphysema accompanies cellular senescence, and the incidence of this disease increases with age (Karrasch, Holz, & Jörres, 2008; Tsuji et al., 2006). Recent studies on the genetic and pharmacological ablation of senescent cells have demonstrated that these cells underlie several diseases (He & Sharpless, 2017). Our results also suggest that the elimination of p19^ARF^‐expressing cells in ARF‐DTR mice protects lung tissues against PPE‐induced emphysema. In contrast to INK‐ATTAC and p16‐3MR mice that express genes conferring drug sensitivity from the *INK4a* promoter (Baker et al., 2011; Demaria et al., 2014), ARF‐DTR mice express the *DTR* gene under the control of the *ARF* promoter/enhancer (Hashimoto et al., 2016). Nevertheless, the administration of DT resulted in the disappearance of not only *ARF* but also *INK4a*, *p21*, and other senescence‐associated genes in lung tissues (Hashimoto et al., 2016). Hence, it is reasonable to conclude that the protective effects of PPE‐induced pathologies are attributed to the ablation of cells expressing those genes.

In addition to emphysema, COPD is frequently associated with an increased number of alveolar inflammatory cells (Taraseviciene‐Stewart & Voelkel, 2008), and PPE‐induced emphysema also causes alveolar inflammation (Houghton et al., 2006). In the PPE‐induced emphysema model, elastin fragments produced by PPE increase macrophage numbers, which secrete elastolytic enzymes such as MMPs (Houghton et al., 2006; Hunninghake et al., 1981). This inflammation is an essential step in the development of PPE‐induced emphysema, and the suppression of alveolar inflammation has been shown to be sufficient at preventing PPE‐induced alveolar collapse (Shapiro et al., 2003; Ueno et al., 2015). In addition, the inactivation of MMP‐12 or neutrophil elastase confers resistance to cigarette smoke‐induced emphysema in mice (Hautamaki et al., 1997; Shapiro et al., 2003). Our results suggest that the presence of p19^ARF^‐expressing cells facilitates the accumulation of macrophages, which occurs at a relatively early stage after PPE challenge. In the absence of p19^ARF^‐expressing cells, the administration of PPE only resulted in a slight increase in the number of BALF cells, which largely consisted of macrophages, as well as in the expression of *MMP‐12*. Senescent cells secrete a series of chemokines and cytokines, thereby promoting the infiltration of macrophages and other immune cells for their clearance; however, this action may also result in adverse effects by inducing local inflammation (Coppé, Desprez, Krtolica, & Campisi, 2010). SASP appears to elicit deleterious effects in several disease models (He & Sharpless, 2017; Watanabe et al., 2017), and its inhibition was shown to be sufficient for preventing senescence‐induced bone loss (Farr et al., 2017). Thus, SASP appears to accelerate alveolar macrophage accumulation upon PPE challenge and the release of tissue‐destructive enzymes, including MMP‐12, which ultimately leads to alveolar collapse.

We used a semi‐genetic method for the elimination of targeted cells; however, as this method is not feasible in humans, the pharmacological targeting of senescent cells is being investigated (Kirkland & Tchkonia, 2015). The senolytic activities and efficacies of some drugs have already been confirmed in mouse disease models (Childs et al., 2016; Farr et al., 2017; Jeon et al., 2017; Ogrodnik et al., 2017; Roos et al., 2016; Schafer et al., 2017). We used the anti‐apoptotic Bcl‐2 family inhibitor, ABT‐263, in the PPE‐induced emphysema model. ABT‐263, or its related molecule ABT‐737, has been shown to alleviate senescence‐associated pathologies (Chang et al., 2016; Childs et al., 2016; Demaria et al., 2017; Yosef et al., 2016; Zhu et al., 2016, 2015 ), but it may not be effective in all cell types (Schafer et al., 2017). In the PPE‐induced emphysema model, an 8‐week treatment with ABT‐263 resulted in a partial decrease in p19^ARF^‐expressing cell numbers, with a concomitant reduction in *INK4a* expression in lung tissues. It currently remains unclear whether ABT‐263 suppresses the accumulation of macrophages in the early stages of PPE treatment and also if ABT‐263 acts through nonsenescent cells in lung tissues or cells in other tissues as ABT‐263 has been shown to induce apoptosis in cells with a high level of Bcl‐family proteins (Lagares et al., 2017). Nevertheless, our results suggest that pulmonary functions were partially maintained in ABT‐263‐treated animals. Further studies are warranted to investigate whether other senolytic drugs are applicable to this disease model.

The major cause of emphysema in humans is cigarette smoking. The PPE‐induced model is highly reproducible but does not capture all features of human emphysema (Antunes & Rocco, 2011). We also observed that cigarette smoking‐induced pulmonary dysfunction was ameliorated by the elimination of p19^ARF^‐expressing cells (data not shown). Hence, our results support the targeting of senescent cells or a certain function of these cells, such as pro‐inflammatory SASPs, as a promising approach for the prevention and suppression of the progression of emphysema. However, it currently remains unclear whether this approach is effective as a treatment for this disease. Alveolar collapse is considered to be irreversible, and, once alveoli are broken, they may not be reconstructed in adult lung tissues. Nevertheless, it may still be possible to partially restore pulmonary function by reinforcing the remaining alveolar walls. It is important to note that lung tissue compliance was almost fully restored in the absence of p19^ARF^‐expressing cells, while the alveolar airspace was only partially reduced. It has not yet been established whether alveoli remodeling may be induced by activating alveolar stem cells or if p19^ARF^‐ or p16^INK4a^‐expressing cell elimination affects tissue stem cells.

## 4 EXPERIMENTAL PROCEDURES

### 4.1 Animals

All animal experiments were approved by and conducted in accordance with guidelines established by the National Center for Geriatrics and Gerontology Animal Ethics Committee. The animals were maintained under specific pathogen‐free conditions, with a 12‐hr light/dark cycle, constant temperature, and ad libitum access to food (CE‐2; CLEA Japan) and water.

Female wild‐type or ARF‐DTR mice (Hashimoto et al., 2016) with the C57BL/6J background were randomly assigned to groups. Hemizygous ARF‐DTR transgenic mice were used for the analysis. Five units of porcine pancreatic elastase (Elastin Products) in 100 μl phosphate‐buffered saline (PBS) were intranasally administered using a standard pipette tip. For the DT treatment, the mice were intraperitoneally injected with 50 μg/kg body weight of DT (SIGMA) at 2‐week intervals. In the senolytic drug treatment, we used a protocol modified from that of Chang et al. (2016). ABT‐263 (Adooq Science) was dissolved in DMSO to obtain a 100 mM stock solution. The stock solution was diluted with vehicle containing 10% ethanol, 30% polyethylene glycol 400%, and 60% phosphatidylcholine and administered to mice via oral gavage at 25 mg/kg body weight per day. The drug was administered for 5 days per cycle for 4 cycles, with a 1‐week interval between cycles.

### 4.2 Morphometry

All histopathological analyses were performed in a blinded manner. The lungs were fixed with Mildform®20N (Wako Pure Chemicals Industries) at 25 cmH_2_O. Paraffin‐embedded tissues were sectioned (5‐μm‐thick) and stained with hematoxylin and eosin. At least eight randomly selected fields per mouse were photographed. Test lines were randomly drawn on the images, and the intercepts with the tissue structure were counted for each line. Airway and vascular structures were eliminated from the analysis.

### 4.3 In vivo imaging analysis

An in vivo luciferase imaging analysis was performed using the IVIS imaging system (Perkin Elmer). Mice were ventrally shaved and anesthetized with isoflurane (Wako Pure Chemicals Industries), and luciferin (VivoGlo; Promega) was intraperitoneally injected according to the manufacturer's instructions. Luciferase activity was monitored 10 min after the luciferin injection. Luminescence was quantified and analyzed using Living Image® software (Perkin Elmer).

### 4.4 Pulmonary function tests (spirometry)

Pulmonary function tests were performed on a FlexiVent system (Scireq) as previously described (Hashimoto et al., 2016; Shalaby, Gold, Schuessler, Martin, & Robichaud, 2010). The mice were euthanized by an intraperitoneal injection of pentobarbital sodium (100 mg/kg of body weight) and connected to the FlexiVent system after tracheotomy. The mice were ventilated at a respiratory rate of 150 breaths/min with a tidal volume of 10 ml/kg against a positive end‐expiratory pressure of 3 cmH_2_O. Deep inflation, Snapshot‐150, Quickprime‐3, and a pressure–volume loop with constant increasing pressure were consecutively performed three times in each mouse. Static lung compliance (Cst) values were calculated by fitting the Salazar‐Knowles equation to the pressure–volume loop. Dynamic lung compliance (Crs) and resistance (Rrs) values were calculated using a single frequency forced oscillation technique. Pressure, flow, and volume signals obtained from the response to a sinusoidal waveform were fit to the single compartment model using a linear regression. Tissue elastance (H) and damping (G) were obtained from respiratory system impedance data using a constant phase model. All parameters were calculated using FlexiVent software.

### 4.5 BALF cell analysis

BALF cells were analyzed as previously reported (Ueno et al., 2015). In brief, BALF cells were prepared with 1 ml of PBS containing 5 mM EDTA, and the cells were collected from BALF by mild centrifugation. The collected cells were attached to glass slides using StatSpin Cytofuge (Beckman Coulter) and subjected to modified Giemsa staining using the Diff‐Quick stain kit (Sysmex).

### 4.6 Real‐time PCR analysis

The total RNA was isolated from lung tissues using the PureLink® RNA Mini kit (Thermo Fischer Scientific) and reverse‐transcribed using the PrimeScript RT reagent kit with a gDNA eraser (TAKARA BIO) according to the manufacturer's instructions. PCR was performed on a Chromo4 PCR system (Bio‐Rad) using the KOD SYBR qPCR mix (TOYOBO). The following primers were utilized for the amplification of specific genes: *ARF*, 5′‐GCCGCACCGGAATCCT‐3′ (sense) and 5′‐TTGAGCAGAAGAGCTGCTACGT‐3′ (antisense); *INK4a*, 5′‐CCCAACGCCCCGAACT‐3′ (sense) and 5′‐GCAGAAGAGCTGCTACGTGAA‐3′ (antisense); *IL‐1β*, 5′‐GAATGCCACCTTTTGACAGTG‐3′ (sense) and 5′‐CTGGATGCTCTCATCAGGACA‐3′ (antisense); *IL‐6*, 5′‐CAAGAAAGACAAAGCCAGAGTC‐3′ (sense) and 5′‐GAAATTGGGGTAGGAAGGAC‐3′ (antisense); *MMP‐3*, 5′‐ACATGGAGACTTTGTCCCTTTTG‐3′ (sense) and 5′‐TTGGCTGAGTGGTAGAGTCCC‐3′ (antisense); *MMP‐9*, 5′‐GCGTCGTGATCCCCACTTAC‐3′ (sense) and 5′‐CAGGCCGAATAGGAGCGTC‐3′ (antisense); *MMP‐10*, 5′‐GAGCCACTAGCCATCCTGG‐3′ (sense) and 5′‐CTGAGCAAGATCCATGCTTGG‐3′ (antisense); *MMP‐12*, 5′‐CTGCTCCCATGAATGACAGTG‐3′ (sense) and 5′‐AGTTGCTTCTAGCCAAAGAAC‐3′ (antisense); *TIMP‐1*, 5′‐GCAACTCGGACCTGGTCATAA‐3′ (sense) and 5′‐CGGCCCGTGATGAGAAACT‐3′ (antisense); *TIMP‐2*, 5′‐TCAGAGCCAAAGCAGTGAGC‐3′ (sense) and 5′‐GCCGTGTAGATAAACTCGATGTC‐3′ (antisense); *GAPDH*, 5′‐AATGGTGAAGGTCGGTGTG‐3′ (sense) and 5′‐GAAGATGGTGATGGGCTTCC‐3′ (antisense); *DTR‐Luc* (fusion), 5′‐TTTAGGTACCATAGGAGAGGAGG‐3′ (sense) and 5′‐CATCTTCCAGCGGATAGAATGGC‐3′ (antisense).

### 4.7 Immunohistochemistry

Paraffin‐embedded tissue sections were deparaffinized, and antigens were retrieved for 5 min in a pressure cooker at 121°C in pH 9.0 antigen retrieval solution (Nichirei Bioscience). For MMP‐12 staining, the sections were incubated with a rabbit monoclonal antibody (1:100 dilution, BS9869M, Bioworld Technology) at room temperature for 60 min. Sites of antibody binding were visualized with the Histofine simple stain MAX‐PO(R) kit (Nichirei Bioscience). 3,3′‐Diaminobenzidine tetrahydrochloride was used as a chromogen, and the sections were counterstained with hematoxylin. For TIMP‐2 staining, a mouse monoclonal antibody (1:100 dilution, 3A4, Santa Cruz Biotechnology) was applied at room temperature for 60 min and visualized with the Histofine mouse staining kit (Nichirei Bioscience). For immunofluorescence, a rabbit polyclonal antibody against p19^ARF^ (1:300 dilution, ab80, Abcam) and a rat monoclonal antibody against fibroblasts (1:50 dilution, ER‐TR7, Santa Cruz Biotechnology) were used. The sections were visualized with Alexa Fluor 488‐conjugated anti‐rat IgG and Alexa594‐conjugated anti‐rabbit IgG and were counterstained with DAPI.

### 4.8 Statistical analysis

A one‐way ANOVA was performed for the comparison of more than two sets of data. When the statistical model was proven to be significant, differences between combinations of the two groups were analyzed using a Tukey–Kramer or Steel–Dwass test. A two‐tailed unpaired Student's *t* test was used for the comparison of two sets of experimental data. The data displayed a normal variance. Significance was represented by asterisks corresponding to *p < 0.05, **p < 0.01, and ***p < 0.001. No blinding was performed, except for histological quantifications. No statistical method was used to select the sample size.

## CONFLICT OF INTEREST

The authors declare that they have no conflict of interests.

## AUTHORS’ CONTRIBUTION

RM performed the majority of the experiments. MS designed the experiments and wrote the manuscript, with contributions from TS. YS and HB contributed to the establishment of the emphysema model. KK and KS performed the immunohistochemical studies.
