# Supplementary figures and images for: Elimination of p19ARF‐expressing cells protects against pulmonary emphysema in mice
Source: Aging Cell. 2018 Jul 30;17(5):e12827. doi: 10.1111/acel.12827 (PMC6156494; doi:10.1111/acel.12827)

Mikawa et al. Figure S1

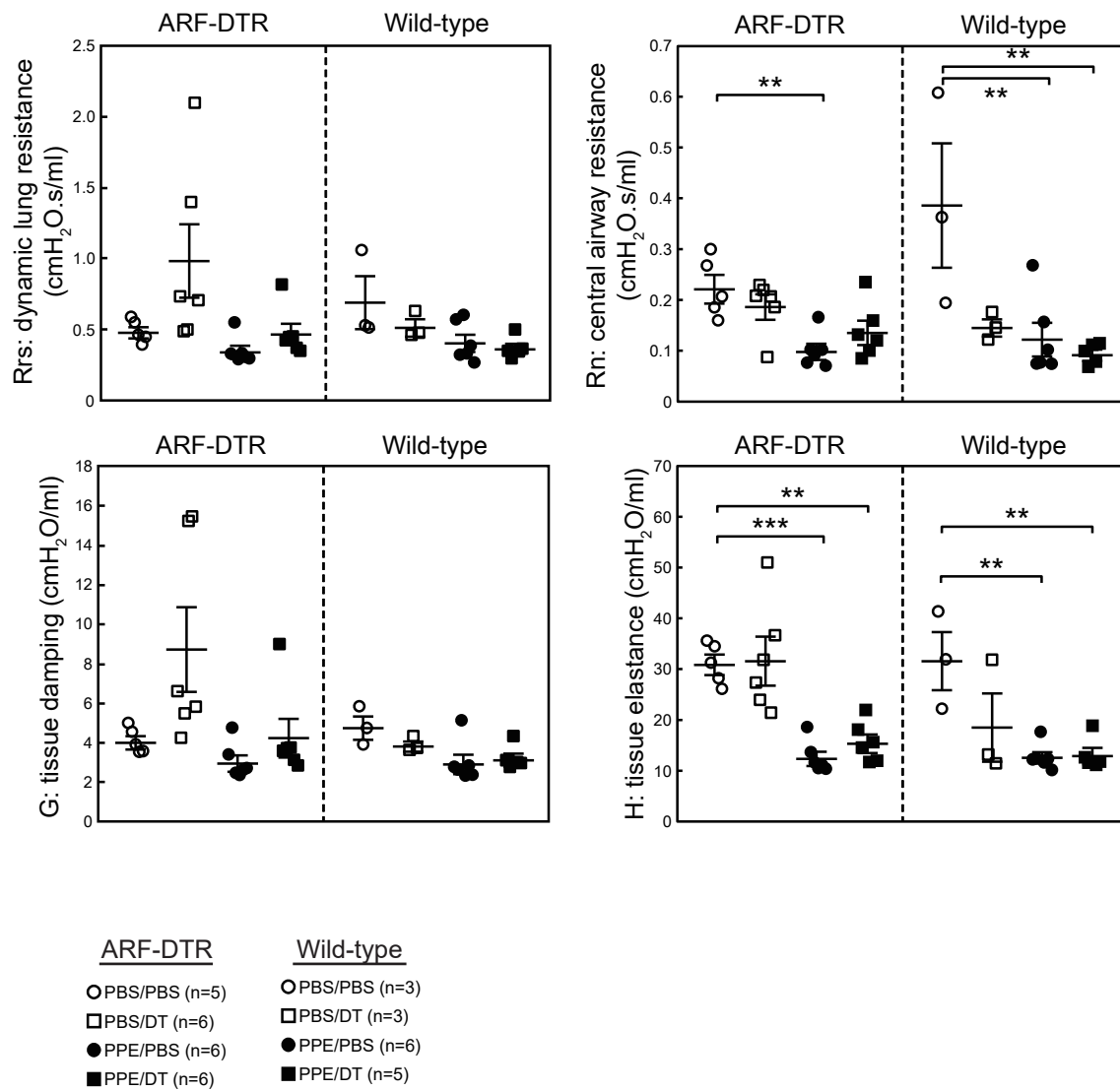

Supplement: Supplementary file 1 [file ACEL-17-e12827-s001.pdf]

Mikawa et al. Figure S2

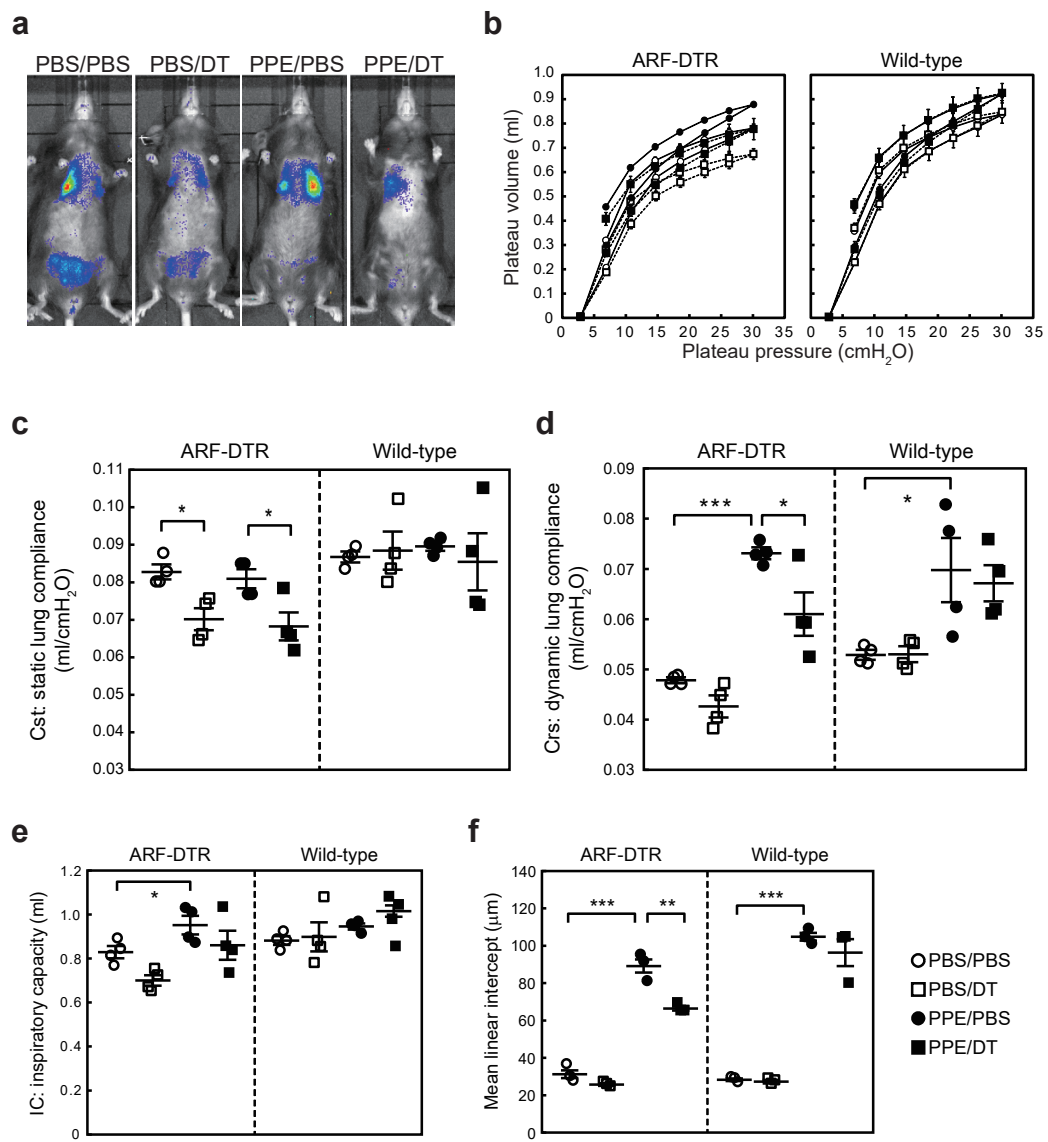

Supplement: Supplementary file 2 [file ACEL-17-e12827-s002.pdf]

Mikawa et al. Figure S3

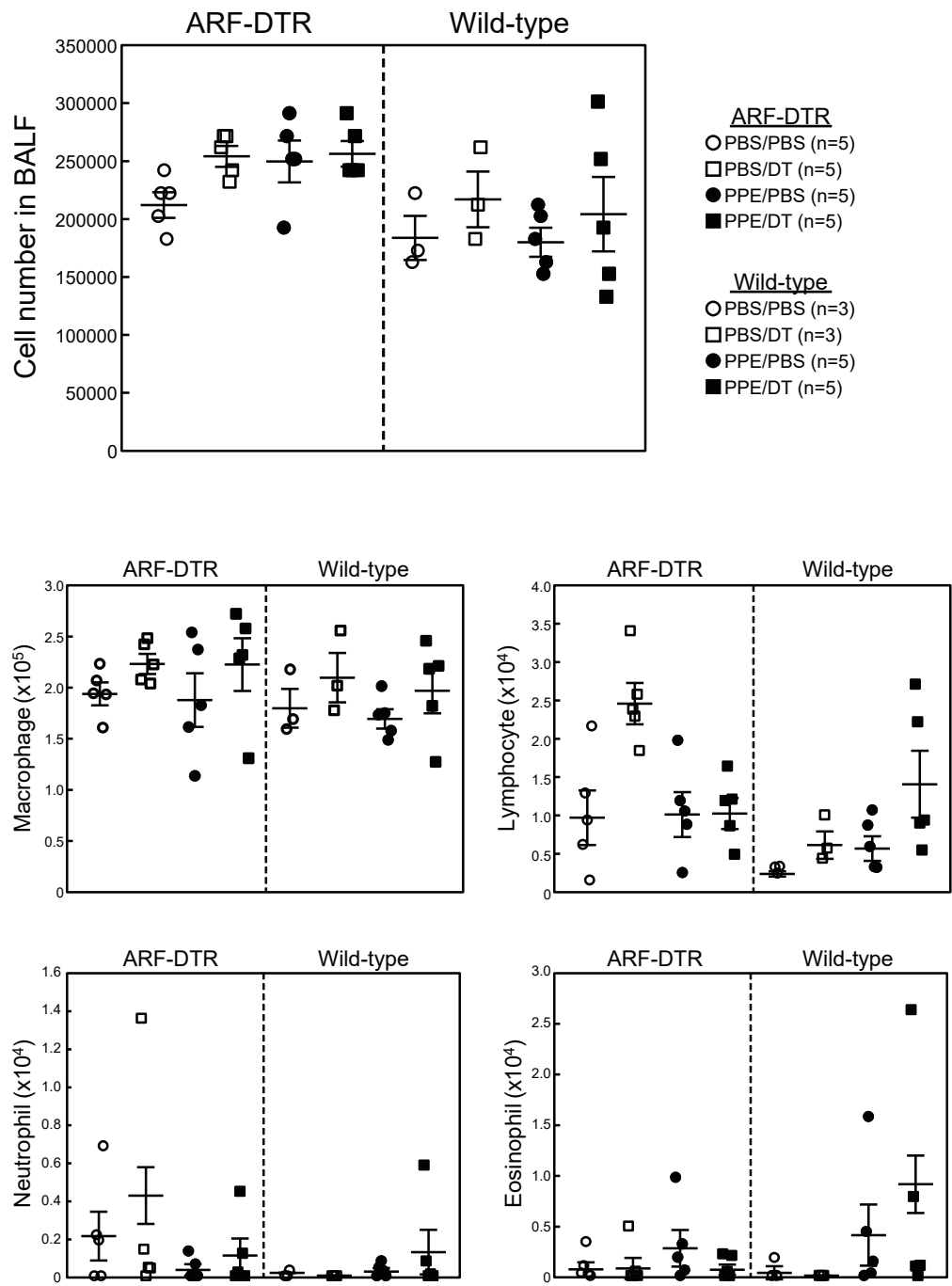

Supplement: Supplementary file 3 [file ACEL-17-e12827-s003.pdf]

Mikawa et al. Figure S4

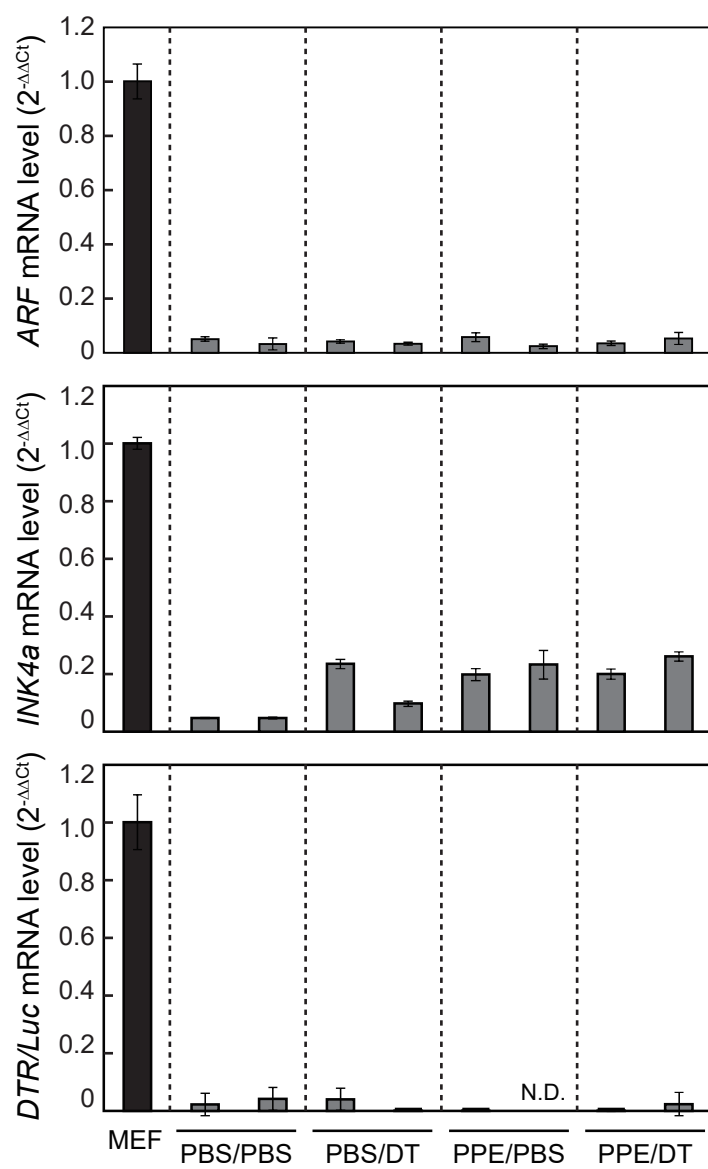

Supplement: Supplementary file 4 [file ACEL-17-e12827-s004.pdf]

**a**

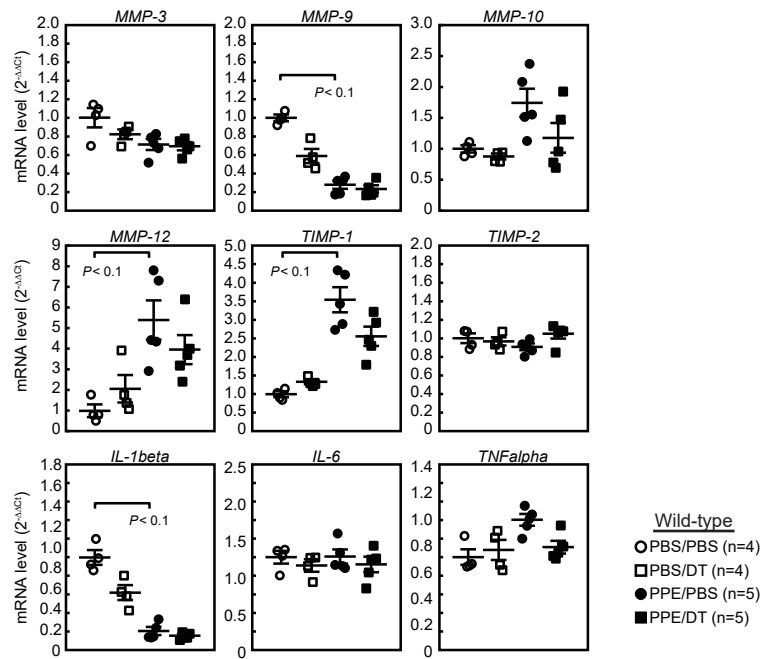

**b**

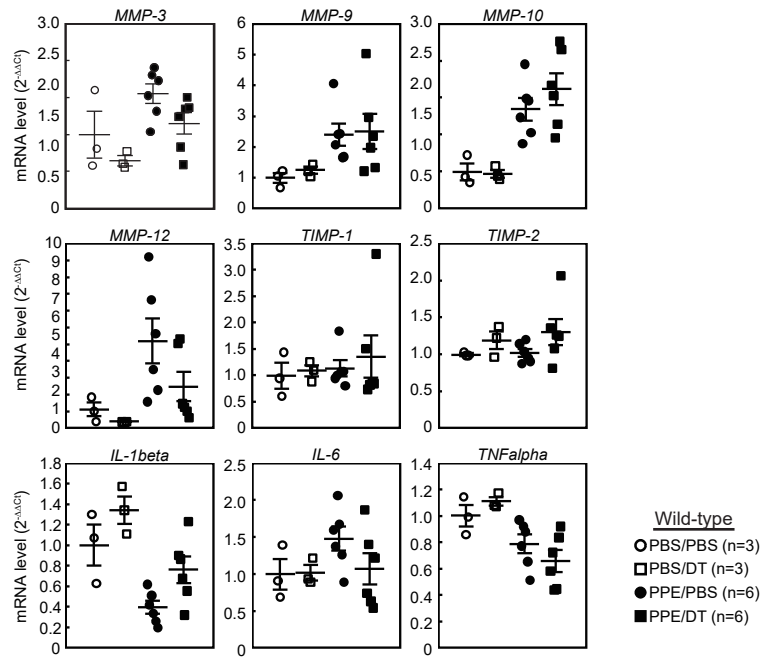

Supplement: Supplementary file 5 [file ACEL-17-e12827-s005.pdf]

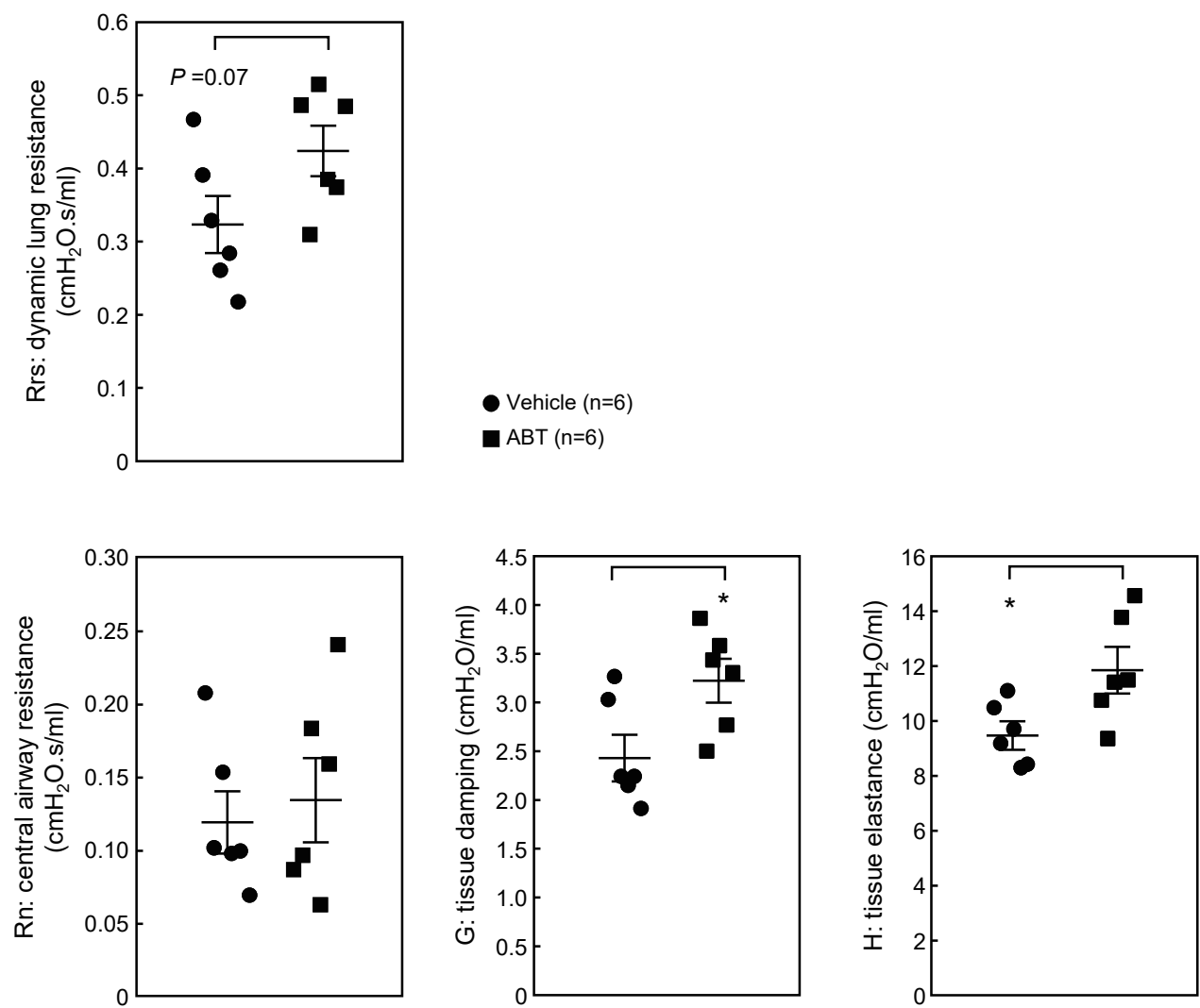

Supplement: Supplementary file 6 [file ACEL-17-e12827-s006.pdf]
